# Potential New Therapies for Pediatric Diffuse Intrinsic Pontine Glioma

**DOI:** 10.3389/fphar.2017.00495

**Published:** 2017-07-25

**Authors:** Wenyong Long, Yang Yi, Shen Chen, Qi Cao, Wei Zhao, Qing Liu

**Affiliations:** ^1^Department of Neurosurgery, Xiangya Hospital, Central South University Changsha, China; ^2^Key Laboratory for Stem Cells and Tissue Engineering, Ministry of Education, Sun Yat-sen University Guangzhou, China; ^3^Department of Histology and Embryology, Zhongshan School of Medicine, Sun Yat-sen University Guangzhou, China; ^4^Center for Inflammation and Epigenetics, Houston Methodist Research Institute, Houston TX, United States

**Keywords:** DIPG, H3K27M, epigenetic therapy, immunotherapy, stem cells, nanoparticles

## Abstract

Diffuse intrinsic pontine glioma (DIPG) is an extensively invasive malignancy with infiltration into other regions of the brainstem. Although large numbers of specific targeted therapies have been tested, no significant progress has been made in treating these high-grade gliomas. Therefore, the identification of new therapeutic approaches is of great importance for the development of more effective treatments. This article reviews the conventional therapies and new potential therapeutic approaches for DIPG, including epigenetic therapy, immunotherapy, and the combination of stem cells with nanoparticle delivery systems.

## Introduction

Diffuse intrinsic pontine glioma (DIPG) is an extensively invasive malignancy of the brainstem with little notable mass effect (Helton et al., 2008; Caretti et al., 2014). These tumors account for >80% of pediatric brainstem gliomas (Guillamo et al., 2001; Salmaggi et al., 2008; Rineer et al., 2010). Children diagnosed with DIPG have a less than 10% 2-year survival rate (Maria et al., 1993; Rubin et al., 1998; Freeman and Perilongo, 1999; Jallo et al., 2006), making DIPG one of the most fatal diseases in children. Although occurring in all age groups, its peak age of onset is 6–7 years (Donaldson et al., 2006), with a second peak in adults aged 20–50 years (median age 34 years) (Landolfi et al., 1998; Guillamo et al., 2001; Rineer et al., 2010). DIPG is equally prevalent in both sexes (male:female = 1:1) (Recinos et al., 2007), and it has been estimated that 100–150 persons are newly diagnosed per year in the United States (Ostrom et al., 2015).

The treatment of DIPG remains unsatisfactory. These tumors cannot be fully removed surgically because of their location and infiltrative nature (Maria et al., 1993; Freeman and Perilongo, 1999). Radiation therapy (RT) remains the standard treatment, but this provides only temporary symptom relief, with no overall survival (OS) benefits. Conventional chemotherapy drugs are also ineffective. Advances in understanding its underlying biology have led to the identification of novel methods of improving patient survival. This review describes traditional and new potential treatments of DIPG.

## Genetic and Epigenetic Alterations of DIPG

**FIGURE 1:**
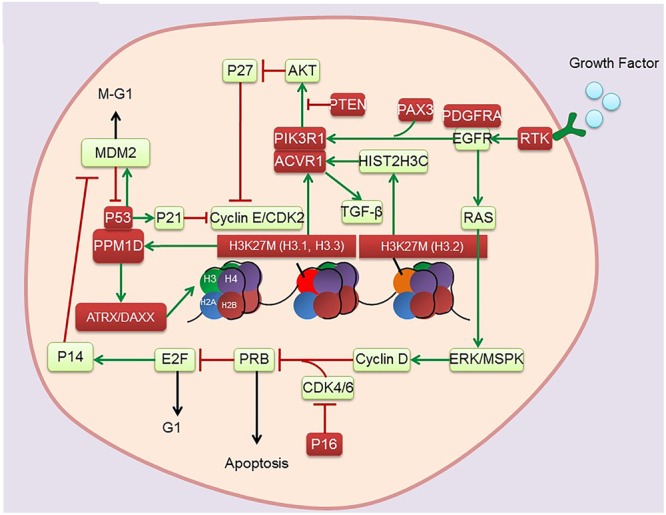
Genetic and epigenetic alterations found in DIPG. Red boxes indicate mutations.

DNA is well organized into nucleosomes containing 147 base pairs, which are wrapped around histone octamers containing two copies each of histones H2A, H2B, H3, and H4 (Esteller, 2005). The N-terminal ends of histones contain lysine (K) and arginine (R) residues, which can be posttranslationally modified (e.g., lysine and arginine methylation, histone lysine acetylation). These modified histones can regulate transcription. Notably, several lysine residues in histone H3, namely, H3K4, K9, K27, K36, and K79, have been found to be methylated and have been comprehensively studied (Barski et al., 2007). Importantly, the missense mutation Lys27 Met (K27M) in the genes encoding histones H3.3 (H3F3A) and H3.1 (HIST3H1B) has recently been identified in pediatric DIPG (Khuong-Quang et al., 2012; Sturm et al., 2012; Wu et al., 2012; Bjerke et al., 2013). Nearly 80% of DIPGs harbor H3K27M mutation, with the presence of mutation inversely correlated with reduced median OS. These findings suggest that epigenetic dysregulation is of great importance for the pathogenesis of DIPGs. Complete understanding of epigenetic alterations in DIPG (**Figure 1** and **Table 1**) presents options for exploring new therapeutic approaches involving the pharmacological targeting of epigenetic pathways.

**Table 1:** Genetic and epigenetic alterations found in DIPG.

Genes mutation in DIPG		Prevalence	Co-occurrence	Epigenetic dysregulation
H3K27M	H3F3A	63% 84%	Co-occurrence with TP53 (75%), PDGFRA (40%), ACVR1 (30%), PPM1D (22%)	Hypomethylation
	HIST1H3B or	21%
	HIST1H3C
TP53		55%	Co-occurrence with H3K27M (59%)	N/A
ACVR1		24%	Co-occurrence with HIST1H3B	Hypomethylation
PDGFRA		16%	Co-occurrence with H3F3A	Hypomethylation
PPM1D		15%	Co-occurrence with H3F3A	Hypomethylation
ATRX/DAXX		9%	Co-occurrence with HIST1H3B (100%)	Chromatin remodeling
MYCN/MYC		7.70%	N/A	Hypermethylation
Others		N/A	N/A	N/A

### Histone Methylation and Chromatin Machinery

Histones deposited during DNA synthesis are the key protein components of chromatin. Selective or reversible modifications of these proteins are responsible for conformational transitions between transcriptionally active and inactive chromatin states. In mammals, three isoforms (H3.1, H3.2, and H3.3) of histone H3 share highly conserved amino acid sequences. H3.1 and H3.2, which are replication dependent, are synthesized and deposited on DNA during S phase, whereas H3.3 synthesis and DNA deposition continue throughout the cell cycle (Maze et al., 2013; Skene and Henikoff, 2013). Posttranslational modifications, such as methylation at the N-terminal of histone H3, play crucial roles in DNA replication, repair, and transcription (Barski et al., 2007). In addition, the extent of methylation of lysine residues (mono-, di-, or tri-methylation) on histone H3 can alter the functional properties of DNA (Broniscer et al., 2010). Notably, methylation of histone H3 lysine 27 (H3K27) is particularly influential in determining the expression of cancer-related genes.

In general, H3K27 methylation status is determined by the methyl-transferase activity of EZH2 and the de-methylation activities of JMJD3/KDM6B and UTX/KDM6A (Agger et al., 2007). However, up to 80% of DIPGs harbor a mutation in canonical H3 variants (H3.1 and H3.3) that encode HIST1H3B or H3F3A, leading to the expression of H3K27M (Buczkowicz et al., 2014; Fontebasso et al., 2014; Taylor et al., 2014) H3K27M has been shown to bind to EZH2, a catalytic subunit of polycomb repressive complex 2 (PRC2), and inhibit PRC2 function. This can result specifically in globally reducing H3K27me3 levels and altering the transcription of various genes, a process that may partially account for the associations of histone H3 mutations with a more aggressive clinical course and poorer OS rates (Lewis et al., 2013).

Mutant IDH1 can directly establish CpG island methylator phenotype_(CIMP) and induce extensive DNA hypermethylation. In addition, mutant IDH1 also increases levels of H3K9me2, H3K27me3, and H3K36me3. Mechanistically, expression of mutant IDH1 results in production of 2-hydroxyglutarate (2-HG), which is the competitive antagonist of 2-Ketoglutaric acid (2-KG). 2-HG subsequently leads to activity inhibition of 2-KG dependent DNA dioxygenases, such as TETs (Turcan et al., 2012). Thus, IDH1 mutations result in the inhibition of histone lysine demethylases, IDH1-mutated tumors tend to be globally hyper-methylated compared with tumors without IDH1 mutations (Fathi and Abdel-Wahab, 2012; Zhang et al., 2014). In contrast to H3K27M tumors, median OS is higher in tumors with than without IDH1 mutations. Thus, strategies opposite to those used to treat hypomethylated DIPGs with H3K27M mutations will be needed to treat hypermethylated DIPGs with IDH1 mutations.

### H3.3-ATRX-DAXX Chromatin Remodeling Complex

ATRX and DAXX constitute two subunits of the H3.3-ATRX-DAXX chromatin remodeling complex at telomeres and other genomic sites. ATRX mutations (loss of function) have been reported in 9% of DIPGs, but are not prevalent in low-grade gliomas in children (Zhang et al., 2013). Moreover, somatic mutations in the ATRX-DAXX complex have been observed to co-occur with K27M mutations with a frequency of co-occurrence reported as 8% in pontine gliomas (Solomon et al., 2016). Interestingly, ATRX mutations are more frequent in children diagnosed with DIPG at an older age (Khuong-Quang et al., 2012). Depletion of ATRX and/or DAXX reduces the incorporation of H3.3 into heterochromatic regions, leading to the destabilization of telomeres and promoting alternative lengthening of telomeres (ALT) (Heaphy et al., 2011). ALT is the predominant mechanism of telomere maintenance in the presence of H3F3A and ATRX in pediatric GBMs (Schwartzentruber et al., 2012). Moreover, H3K27M upregulation of HIPK1 may increase the translocation of DAXX, which may in turn alter chromatin and telomere structure, causing aberrant gene expression in DIPGs (Saratsis et al., 2014). Gain of function in the H3.3-ATRX-DAXX axis may also guide drug development for DIPG.

### H3K27M Crosstalk with Histone Acetylation and DNA Methylation

Current analysis of histone methylation in DIPGs cannot fully determine the relationships between histone modifications and DIPG development. Over-acetylation of H3K27 was found to be mutually exclusive of K27 mutation. Thus, increased K27 acetylation was found to be accompanied by decreased K27me3 in K27M mutant glioma cells (Lewis et al., 2013), a finding confirmed in the *Drosophila melanogaster* model (Herz et al., 2014). Furthermore, PRC2 loss-of-function phenotypes in the Drosophila model resembled the reduced H3K27 methylation and depression of PRC2 target genes in DIPGs. These findings suggested that K27 acetylation may also be responsible for glioma formation and that K27 methylation and acetylation should be studied in juxtaposition. Investigations regarding the relationships between K27 modifications and tumor development should therefore include screening for enzymatic activities responsible for all types of K27 modifications. The elevated level of H3K27 acetylation observed in DIPGs suggests that inhibition of histone deacetylase (HDAC) may potentially benefit patients.

In addition to histone modifications, DNA methylation plays a key role in coordinating gene expression and chromatin remodeling in brain tumors. DNA methylation, which occurs at cytosine residues of CpG dinucleotides, is usually associated with gene silencing (Schubeler, 2015). DNA methylation profiles across all tumor sites in DIPG tissues were shown to be associated with alterations in a specific histone 3 variant (Fontebasso et al., 2014). In mammals, DNA methylation occurs primarily at the 5-position of cytosines (5mC) in CpG dinucleotides. Methylated cytosines at gene promoters are usually associated with transcriptional silencing. Notably, Ten Eleven Translocation (TET) enzymes can convert 5mC to 5hydroxymethylcytosine (5hmC) dependent on alpha-ketoglutarate (α-KG). Previous studies revealed that loss of 5mC leads to a redistribution of PRC2 complexes, indicating that 5mC could affect interaction between PRC2 and chromatin. Aberrant recruitment of PRC2 to DNA associated with 5hmC may shift PRC2 away and promote an active transcriptional state. Low levels of H3K27me3 and 5mC and higher levels of 5hmC were found to be more frequent in DIPGs with H3K27M than in extrapontine GBMs. Generally, high levels of 5hmC have been shown to be a feature of terminally differentiated cells (Haffner et al., 2011) and to be associated with a less aggressive phenotype (Orr et al., 2012). However, dysregulation of histone and cytosine methylation is unique to DIPGs, suggesting putative crosstalk between histone and DNA methylation pathways, thereby altering transcriptional activity (Bender et al., 2013). Thus, the finding, that DNA methylation profiles are associated with the K27M mutation regardless of tumor location, supports its role in driving the epigenetic phenotype and establishes a foundation for treatment with specific inhibitors of DNA methylation (Morales and Kieran, 2017).

### Bromodomain and Extraterminal (BET) Associated Genes Regulated MYCN Pathways

Bromodomain and extraterminal (BET) family proteins are associated with transcriptional activation through their interactions with acetylated chromatin, as well as playing key roles as epigenetic regulators (Dhalluin et al., 1999). BET proteins regulate the expression of certain significant oncogenes including those genes involved in the cell cycle and apoptosis pathways. Elevated H3K27 acetylation has also been associated with increased levels of bromodomain containing proteins 1 (BRD1) and 4 (BRD4) (Herz et al., 2014). Measurement of CpG island methylation allows identification of a subgroup of DIPGs with high-level amplification of the MYCN pathways in DIPG (Buczkowicz et al., 2014; Taylor et al., 2015). Despite difficulties in directly targeting MYCN, the bromodomain-mediated inhibition of MYCN attenuated tumor growth and induced apoptosis, conferring a survival advantage in three *in vivo* models of neuroblastoma (Puissant et al., 2013). Due to the potential of BET as an epigenetic target, small molecule inhibitors of BET proteins are being broadly screened (Wadhwa and Nicolaides, 2016). It is worth noting that the majority of the heterotypic H3K27M-K27ac nucleosomes colocalize with bromodomain proteins at the loci of actively transcribed genes. Piunti et al. (2017) found that treatment of DIPG cells with BET bromodomain inhibitor JQ1 significantly suppressed the tumourigenicity *in vivo*, thus identifying this class of compounds as a novel epigenetic therapy to overcome DIPG.

## Progess in Traditional Treatments for DIPG

Diffuse intrinsic pontine glioma is almost invariably fatal with a mean OS of 9–12 months from the time of diagnosis. RT provides temporary symptom relief but no OS benefits. Several types of adjuvant therapy, such as small molecules targeting tumor proliferation, apoptosis, the cell cycle, angiogenesis, DNA repair, and radiation sensitizers have been studied, but none has had any promising impact on patient outcomes. Although recent advances in understanding the anatomic details of brainstem structures and the availability of neuroimaging monitoring may warrant biopsy at diagnosis and subtotal resection in some patients, surgical resection remains uncommon. Combinations of traditional therapy with epigenetic therapy, immunotherapy and nanotechnology show exciting therapeutic potential.

### Radiation Therapy

Treatment with a total radiation dose of 54–60 Gy over 6 weeks was found to result in temporary symptom relief in patients with pediatric DIPG, as well as delaying tumor progression in about 70–80%. Re-irradiation significantly increases risks of radiation toxicity at doses >64 Gy (Bartels et al., 2011) without improving survival outcomes (Fontanilla et al., 2012; Susheela et al., 2013). Because DIPG biology appears to shift from initial diagnosis to tumor progression (Wolff et al., 2012), re-planning of therapy may be warranted (Chris Beltran, 2011).

Treatment of 16 pediatric brainstem glioma patients with a combination of interferon-beta, nimustine (ACNU), and radiation (IAR therapy) resulted in a median OS of 15.7 months, a marked improvement compared with the natural course of this disease (Wakabayashi et al., 1992). No further clinical benefit was achieved with alternative radiation strategies or from combinations of radiation with radiation sensitizers (Robison and Kieran, 2014; Roos and Smith, 2014), such as temozolomide (Sirachainan et al., 2008), capecitabine (Kilburn et al., 2013), panobinostat (Hennika et al., 2017), and tipifarnib (Haas-Kogan et al., 2011). A Children’s Oncology Group phase I study indicated that depletion of DNA repair enzymes with motexafin gadolinium (MGd)-enhanced radiation sensitivity about twofold (Bradley et al., 2008). However, this finding was not confirmed in a subsequent Children’s Oncology Group phase II Study (Bradley et al., 2013). Other radiation sensitizers remain to be tested, including the PARP1 inhibitor niraparib (Chornenkyy et al., 2015), the bromodomain inhibitor JQ1, the NOTCH signaling inhibitor MRK003 (Taylor et al., 2015), the CDK4/CDK6 inhibitor PD-0332991 (Barton et al., 2013), and the G2 cell-cycle checkpoint WEE1 kinase inhibitor MK-1775 (Caretti et al., 2013). Although *in vitro* and *in vivo* results indicate that these compounds may be effective radiation sensitizers for DIPG, multi-center clinic trials are required to validate these findings.

### Targeted Therapy

The multistep development of human tumors includes the gain of eight biological capabilities: sustained proliferative signaling, evasion of growth suppressors, resistance to cell death, enabling of replicative immortality, induction of angiogenesis, activation of invasion and metastasis, reprogramming of energy metabolism and evasion of immune system destruction (Hanahan and Weinberg, 2011). Since the introduction of mechanism-based molecular targeted therapies to treat DIPG, more than 250 clinical trials aimed at different biological capabilities of DIPG have been initiated. The gene encoding the receptor tyrosine kinase (RTK) platelet-derived growth factor receptor alpha (PDGFRA) is one of the most frequently amplified genes in DIPGs (Paugh et al., 2010, 2013; Zarghooni et al., 2010; Barrow et al., 2011; Puget et al., 2012), with activating mutations in *PDGFRA* observed in 10% of DIPGs (Balss et al., 2008). Agents targeting PDGFR, such as imatinib and dasatinib, have exhibited low antitumor effects in clinical trials (Truffaux et al., 2015). The gene encoding epidermal growth factor receptor (EGFR) is also overexpressed in pediatric brain tumors (Bredel et al., 1999; Gilbertson et al., 2002), suggesting that targeting of EGFR may have benefits for patients. Trials of anti-EGFR drugs, such as nimotuzumab (Bartels et al., 2014), gefitinib (Pollack et al., 2011), and erlotinib (Geoerger et al., 2010), have benefits in a small subset of DIPG patients. Other trials have targeted DNA repair, using the PARP1 inhibitors veliparib, olaparib, and niraparib (Chornenkyy et al., 2015), the anti-MGMT agent O6-benzylguanine (Warren et al., 2012), and MGd. Additional trials have tested the multi-kinase inhibitor BMS-754807 (Halvorson et al., 2015), the CDK4/CDK6 inhibitor PD-0332991, the WEE1 kinase inhibitor MK-1775, the antiangiogenic agent bevacizumab, a monoclonal anti-VEGF antibody (Liu et al., 2009; Gururangan et al., 2010), and the farnesyltransferase inhibitor tipifarnib (Haas-Kogan et al., 2011). To date, however, none of these trials has shown efficacy in DIPG. These treatment failures may be caused by the presence of drug efflux transporters, the ability of the tested drugs to cross the blood–brain barrier (BBB), or other resistance mechanisms (Veringa et al., 2013). Alternative methods of drug delivery, such as nanoparticle deliver systems, may offer new possibilities for the treatment of pediatric DIPG.

### Surgical Biopsy and Resection

The brainstem region has long been considered a “forbidden area” for surgery. The shortage of readily available DIPG tissue samples for molecular analysis has hindered the investigation of these tumors and their molecular biology. Due to the potential risks of the surgical procedure and poor benefit for patients, biopsy of DIPG was abandoned by the majority of neurosurgical teams in the last 20 years. However, with the development of novel molecular genetic techniques and existence of various molecular signatures which indicated for different therapeutic schemes and agents, the role of stereotactic biopsy during the treatment of DIPG was gradually refocused in the recent years (Puget et al., 2015; Carai et al., 2017). Meanwhile, studies of brainstem anatomy have revealed 12 “safe entry zones” in the brainstem, including the perioculomotor (Bricolo et al., 1991); lateral mesencephalic sulcus; suprafacial (Kyoshima et al., 1993), interfacial (Bricolo and Turazzi, 1995), and lateral (Lawton et al., 2006) sulcus limitans; periolivary; posterior median sulcus; infraclavicular and supraclavicular areas; and the peritrigeminal, infrafacial, and supratrigeminal zones (Cavalheiro et al., 2015). However, it is nearly impossible to surgically resect intrinsic gliomas completely due to diffuse malignant infiltration into white matter tracts; hence, surgery is still not recommended approach for pediatric DIPG.

## New Potential Treatments for DIPG

### Epigenetic Therapy

**FIGURE 2:**
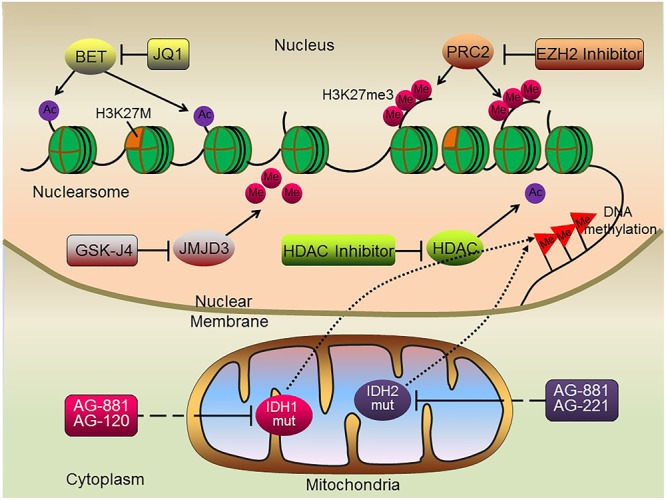
Schematic illustration of potential epigenetic therapies for DIPG. Histone methylase and demethylase inhibitors, histone deacetylase inhibitors, BET protein inhibitors and DNA methylation inhibitors may all serve as potential therapeutic targets suppressing tumorigenesis of H3K27M DIPGs.

Growing evidence suggests that epigenetic alterations, either alone or in combination with gene mutations, are significantly associated with DIPG development and progression. The high frequency of H3K27M mutations in DIPG, resulting in the marked reduction of H3K27me3 levels, has led to the development of novel therapeutic strategies targeting enzymes responsible for chromatin modifications (**Figure 2**).

#### Histone Methylase and Demethylase Inhibitors

Up to 80% of DIPGs harbor a mutation in H3K27M (Schwartzentruber et al., 2012; Wu et al., 2012; Buczkowicz et al., 2014; Fontebasso et al., 2014; Taylor et al., 2014), resulting in global reductions in H3K27me3 levels (Lewis et al., 2013). H3K27me3 levels are regulated by the H3K27 methylating enzyme EZH2 and the de-methylating enzyme JMJD3/KDM6B. EZH2 was found to be highly expressed in a variety of solid tumors, including colon (Tan et al., 2007) and prostate (Varambally et al., 2002, 2008) cancers and hematological malignancies (Morin et al., 2010, 2011). Moreover, high EZH2 expression correlated with poor median OS (Helin and Dhanak, 2013), indicating that EZH2 is a potential therapeutic target for H3K27M-mutant DIPG. However, treatment of pediatric GBM/DIPG cells harboring either a H3.3 mutation or a H3 wild type with the EZH2 inhibitor EPZ-6438 had little effect on these cells (Wiese et al., 2016), indicating that EZH2 inhibition alone might be ineffective in patients with DIPG. In contrast, treatment of DIPGs having a H3K27M mutation with the EZH2 inhibitor tazemetostat resulted in global loss of H3K27me3, several genes retained H3K27me3, with residual PRC2 activity required for the proliferation of H3K27M-expressing DIPGs (Mohammad et al., 2017). The difference between these two studies may have been due to sample bias, indicating a need for additional studies.

As H3K27M DIPG cells with reduced di- and trimethylation (H3K27me2 and H3K27me3) are transcriptionally more active, another potential strategy has focused on inhibiting the Jumonji-domain demethylase JMJD3, a key enzyme responsible for the demethylation of H3K27 (Lewis and Allis, 2013). Treatment of H3K27M-expressing DIPG cells and brainstem glioma xenografts with the GSK-J4, an inhibitor of JMJD3 demethylase, resulted in complete growth inhibition, along with increased K27 methylation and a subsequent decrease in gene expression (Hashizume et al., 2014). This effect was further enhanced by panobinostat (Bagcchi, 2015; Grasso et al., 2015), an agent approved by the FDA for other indications. At present, clinical trials are being planned to test single and combination therapy in patients with DIPG (Staedtke et al., 2016).

#### Histone Deacetylase Inhibitors

Histone deacetylase inhibitors prevent histone deacetylation, thereby facilitating an open chromatin structure and resulting in gene activation. The pan-HDAC inhibitor, vorinostat, showed high activity against high-grade gliomas and other pediatric central nervous system (CNS) tumors (Milde et al., 2011; Kitange et al., 2012). Screening of DIPG cells with 83 small molecules identified a multi-HDAC inhibitor panobinostat, which has been developed for the treatment of various cancers (Ellis et al., 2008). This agent showed the highest anti-DIPG activity *in vitro*, an order of magnitude better than vorinostat (Grasso et al., 2015). Indeed, a dose-dependent increase in global H3 acetylation and H3K27 trimethylation following panobinostat treatment suggested that this drug partially rescued the H3K27M-induced hypotrimethylation phenotype (Grasso et al., 2015). Increased H3K27 trimethylation revealed that poly-acetylation of the H3 N-terminal tail can rescue K27M-induced inhibition of the PRC2 (Brown et al., 2014) and subsequently normalize the K27M gene expression signature, further reducing the expression of genes targeted by the oncogene MYC (Grasso et al., 2015). In addition to panobinostat being tested in clinical trials of adults with brain tumors (NCT01324635, NCT00848523), the side effects and optimal dose of panobinostat are currently being tested for the treatment of younger patients with DIPG (NCT02899715, NCT02717455). Concomitant treatment with panobinostat and reirradiation for patients with DIPG showed promising effects (Wang et al., 2017). Panobinostat was shown to reduce the expression of oncogenes, and to increase global H3 acetylation and H3K27 trimethylation, in DIPG cells. A combination of panobinostat and GSK-J4 showed a synergistic effect against DIPG cells, indicating a need for studies of the anti-tumor activities exhibited by multiple histone modifiers.

#### DNA Methylation Inhibitor

Approximately 25% of brainstem gliomas harbor IDH mutations (Zhang et al., 2014), leading to global hyper-methylation and suggesting that hypo-methylating agents may be therapeutically effective in patients with DIPG. Two preclinical studies have shown that DNA hypo-methylating agents, including decitabine and 5-azacytidine, suppressed the growth of IDH1-mutant tumor cell lines (Borodovsky et al., 2013; Turcan et al., 2013). Intraperitoneal injection of 5-azacytidine into nude mice bearing subcutaneous xenografts derived from an IDH1-mutant anaplastic astrocytoma specimen resulted in significant tumor regression 14 weeks later, with no signs of re-growth 7 weeks subsequently, despite discontinuation of therapy. These results have implications for phase I pharmacokinetic trials assessing the ability of DNA hypo-methylating drugs like 5-azacytidine to cross the BBB and reach tumor sites at high enough concentrations and with acceptable side effects. AGI-5198 and ML309 are specific inhibitors of IDH1 R132H, reducing 2-HG levels and significantly decreased the growth of IDH1^R132H^-expressing glioma cells *in vitro* and human glioma xenografts (Davis et al., 2010; Rohle et al., 2013). Mutant-selective inhibitors of IDH1 (AG-120), IDH2 (AG-221) and both IDH1/2 (AG-881) have entered phase I clinical trials (NCT02073994, NCT02273739, and NCT02481154, respectively) (Chen et al., 2016). Interestingly, a phase I trial of AG-120, an oral inhibitor of mutant IDH1, resulted in stable disease in 10 of 20 patients with IDH1 mutant glioma, with a 6-month clinically beneficial response rate of 25% (Chen et al., 2016).

#### BET Protein Inhibitors

The acetylation of lysine residues at the N-terminal of histones is associated with activation of transcription through opening of chromatin architecture (Marushige, 1976). This allows for assembly of transcriptional complexes by recruiting BET family proteins such as BRD2, BRD3, and BRD4 (Delmore et al., 2011). The finding, that BET family proteins play a critical role in transcriptional activation and have oncogenic potential, suggested that BET proteins may be potential therapeutic targets in cancers, Several small molecule BET protein inhibitors were therefore developed for testing. One is JQ1, a histone binding module inhibitor that competitively binds to bromodomains with high potency and specificity. JQ1 displaces the BRD4 fusion oncoprotein from chromatin, inducing cell-cycle arrest and initiating apoptosis (Filippakopoulos et al., 2010), suggesting that JQ1 may have wide use in cancer treatment.

Medulloblastoma, the most common malignant brain tumor in children, consists of at least four distinct subtypes, including wingless (WNT), sonic hedgehog (SHH), and groups 3 and 4 (Cho et al., 2011; Northcott et al., 2011). Amplifications of MYC and MYCN are frequently found in subtypes with the poorest prognosis (Northcott et al., 2012; Bandopadhayay et al., 2014). Notably, MYC protein, a notoriously difficult direct target for novel drug development, together with SHH, is locked within the frame of BET bromodomain inhibition (Leary and Olson, 2012; Tang et al., 2014). JQ1 treatment of medulloblastoma patients and genetically engineered mouse model-derived medulloblastoma cell lines and xenografts with MYC or MYCN amplifications was found to suppress MYC expression and MYC-associated transcriptional activity, thereby reducing cell viability in medulloblastomas (Bandopadhayay et al., 2014).

Recent molecular and proteomic analyses of autopsy specimens have identified key genetic alterations in DIPG, as well as distinct DIPG subgroups based on activation of the SHH and MYCN pathways (Saratsis et al., 2014). The high levels of MYCN and SHH in DIPG led to testing the efficacy of sequential therapy with the MYCN and SHH-targeted bromodomain inhibitor JQ1, and the NOTCH-targeted gamma secretase inhibitor MRK003. Dual targeting with JQ1 and MRK003 inhibited DIPG growth and induced apoptosis, suggesting that this therapeutic regimen may be effective (Taylor et al., 2015). More extensive preclinical trials of BET protein inhibitors, as well as the translation of promising results to phase I/II clinical trials in patients with DIPG, are required.

### Immunotherapy

**FIGURE 3:**
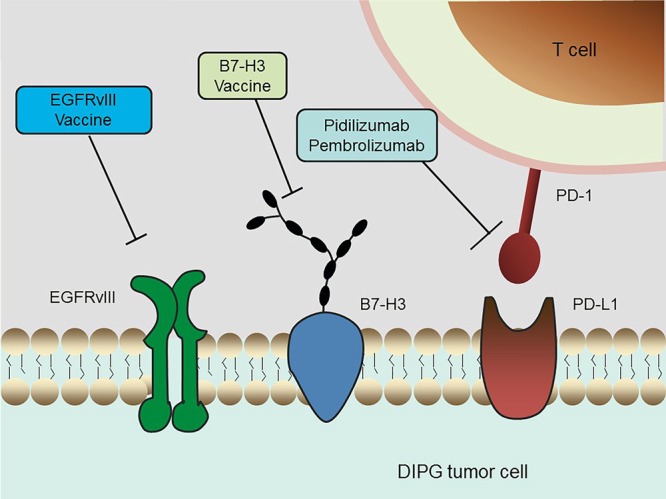
Schematic overview of current immunotherapeutic strategies in DIPG. Multiple immunotherapy methods that are undergoing clinical trials in DIPG, including an EGFRvIII peptide vaccine, a B7-H3 vaccine and anti-PD-1 immune checkpoint inhibitors, are depicted.

Immunotherapy is rapidly becoming the newest pillar of malignancy treatment, with the potential to harness the potency and specificity of the host immune system. The CNS was historically considered an immune-privileged site because it lacks a conventional lymphatic drainage system (Carson et al., 2006; Vauleon et al., 2010). However, the recent discovery of a lymphatic system within the CNS and novel insights into the mechanisms by which tumor cells evade surveillance by the immune system have stimulated studies of immunotherapy in brain cancers like DIPG (Louveau et al., 2015; Preusser et al., 2015) (**Figure 3**).

Several studies focused on the identification of DIPG-specific antigens and evaluated their application to vaccine production. EGFR variant III (EGFRvIII) is one of the most common mutations in GBM. The tumor cell selectivity of EGFRvIII was utilized to develop an immunotherapy vaccine for GBM (Li et al., 2010; Staedtke et al., 2016). EGFRvIII was found to be expressed in nearly 50% of the DIPG samples tested, a percentage high enough for therapeutic targeting (Li et al., 2012). A phase I trial is currently testing treatment with the EGFRvIII peptide vaccine after conventional RT. B7-H3, or CD276, is a type I transmembrane glycoprotein belonging to the B7-CD28 family (Chapoval et al., 2001). This glycoprotein is present in most neuroepithelial tumors, but not in normal neurons, makes this antigen an attractive therapeutic target in CNS tumors. The immunoreactivity of B7-H3 in DIPG specimens was confirmed immunohistochemically (Zhou et al., 2013). The level of expression of B7-H3 was significantly higher in DIPG than in normal brain samples, indicating that this tumor specific antigen (TSA) may serve as a potential therapeutic target in antibody-based immunotherapy. In addition, the gene encoding the neuropeptide prepronociceptin (PNOC) was found to be the most up-regulated gene in pediatric brainstem gangliogliomas relative to non-brainstem gangliogliomas, suggesting that PNOC may have diagnostic significance and that it can be potentially used as an antigen for immunotherapy (Chan et al., 2012).

The immune checkpoint molecules, which are responsible for preventing immune over-activation, also play a key role in antitumor immunity. Cancer cells have developed a particular strategy to up-regulate the expression of such immunosuppressive factors in order to circumvent the host immune response. To date, immune checkpoint inhibitors that can access the blockage of CTLA-4 or PD-1/PD-L1 have been utilized in clinical trials of nearly all types of tumors, showing convincing efficacy in a series of malignancies (Snyder et al., 2014; Le et al., 2015). For example, the humanized antibodies pidilizumab and pembrolizumab, both of which target and inhibit PD-1, are currently undergoing clinical evaluations in DIPG. The clinical trial of pidilizumab is a Phase I/II (NCT01952769) study, whereas the ongoing trial of pembrolizumab has been temporarily suspended due to severe adverse reactions, such as endocrinological, hepatic, gastrointestinal, and dermatological toxicities (NCT02359565). Additional efforts are required to evaluate the safety and efficacy of immune checkpoint inhibitors in the treatment of DIPG.

### Nanoparticle Delivery Systems

**FIGURE 4:**
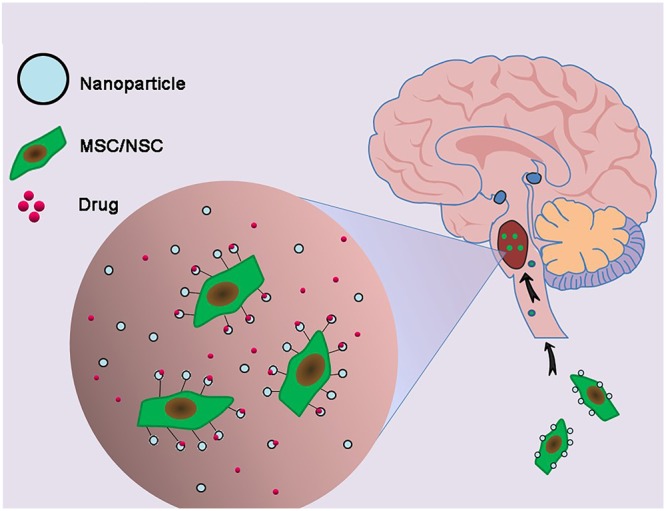
Combination of genetically engineered stem cells and nanotechnology in the treatment of DIPG. The glioma cells-homing behavior of neural stem cells (NSCs) and mesenchymal stem cells (MSCs) was utilized to deliver nanoparticles carrying drugs to DIPG tumor.

Chemotherapy, even with drugs effective *in vitro*, may be unsuccessful *in vivo* due to their inability to penetrate the extremely selective BBB. Moreover, it is difficult to attain a concentration around the tumor site high enough to kill infiltrative tumor cells without damaging ambient normal brain tissues (Pardridge, 2005). Even when enriched within glioma tissues, chemotherapy agents may experience uneven intratumoral distribution due to their entrapment in the extracellular matrix space surrounding neoplastic cells or within intratumoral necrotic pockets, blunting their effects on targeted infiltrative areas. These limitations, however, may be overcome by using stem cell carriers loaded with nanoparticle delivery systems (**Figure 4**).

The development of nanoparticles, based upon stable elements, polymer nanoparticles and organic nanoparticles, is underway for the treatment of brain tumors, including DIPGs. Trial and error based alterations in their size, composition, and surface chemistry, can result in the development of nanoparticles as a universal platform with multifunctional capabilities, enabling them to meet the different requirements of drug delivery systems (Feng et al., 2011). The glioma cell homing behavior of neural stem cell (NSCs) and mesenchymal stem cells (MSCs) (Benedetti et al., 2000; Wu et al., 2008; Ahmed et al., 2011) can be explored in designing therapeutic carriers. Traditional gene therapy for glioma, relying on the use of viral vectors as transporters to deliver the desired genes to cell carriers (Tyler et al., 2009; Ahmed et al., 2011), has several drawbacks, including infection related cell damage and immune response issues (Everts et al., 2006). Nanoparticles bound to DNA escape these limitations and have shown perspectives for gene delivery into stem cell carriers. For example, polyethylenimine (PEI)-DNA coated silica nanoparticles were developed for gene delivery into hMSCs, with 75% of hMSCs taking up these particles (Park et al., 2010). Delivery of the hVEGF gene to hMSCs using biodegradable poly beta-amino ester nanoparticles resulted in enhanced VEGF production by these cells (Yang et al., 2010). Although these studies did not specifically evaluate targets for glioma treatment, the concept of nanoparticle-mediated gene delivery to stem cells may be of value if anti-glioma genes can be integrated into nanoparticle transporters. Genetically engineered human adipose tissue-derived mesenchymal stem cells (hAT-MSCs) encoding the tumor necrosis factor-related apoptosis-inducing ligand (TRAIL) were found to control brainstem gliomas, indicating that of non-virally engineered hAT-MSCs are safe and effective against brainstem gliomas and showing that stem-cell-based targeted gene therapy may be clinically applicable (Choi et al., 2010). At present, various multiple nanoparticle formulations are being investigated for the treatment of DIPGs (Bredlau et al., 2017).

## Future Directions

Diffuse intrinsic pontine gliomas are malignant tumors with epigenetic characteristics, including histone methylation, histone acetylation, DNA methylation and BET family proteins, indicating that agents targeting epigenetic factors are of great importance in treating DIPG. Understanding the epigenetic landscape of DIPG opens up the opportunity for epigenetic modifiers, which could potentially shift the active genome of this deadly tumor into a silent and regulated state. Indeed, epigenetic modifying therapies are emerging as the most promising class of agents for DIPG. Additional studies are also needed to evaluate the anti-tumor activities resulting from the simultaneous inhibition of multiple epigenetic modifications, as none of those epigenetic alterations can be studied in isolation. Identification of DIPG patient-specific molecular signatures and markers, along with further optimization of epigenetic regulator drugs, will be a major challenge in the development of treatments for DIPG. These efforts may lead to agents with therapeutic benefits in stratified patient populations.

Diffuse intrinsic pontine gliomas show poorer clinical responses to agents that are more effective against other glioma types, suggesting that DIPG is a highly heterogeneous group of tumors that differ in cellular origin and pathogenesis. Efforts are therefore needed to treat tumors with many different molecular genetic alterations located in a site with limited access to therapy. Previous treatment failures may be attributed to several factors, including inadequate drug levels within the tumor due to inability to cross the BBB; testing of single agents or agents with signal pathway redundancies, leading to drug resistance; lack of patient pre-selection; and lack of biomarkers to assess response and off-target effects. Nanotechnology may overcome those limitations and result in improved patient survival rates.

Expedited clinical trials addressing specific targets in small numbers of patients being treated at tertiary medical centers will facilitate the development of larger multicenter randomized clinical trials. In addition, more specific and higher affinity antibodies to tumor antigens that can easily enter the brainstem need to be identified. Thus, combinations of epigenetics, immunotherapy, and nanotechnology may identify models for curing pediatric DIPG.

## Author Contributions

WL and YY wrote the manuscript and drew the figures. SC revised the manuscript and modified the figures. QC revised the manuscript according to reviewers’ comments. WZ and QL wrote the manuscript and supervised all the work.

## Conflict of Interest Statement

The authors declare that the research was conducted in the absence of any commercial or financial relationships that could be construed as a potential conflict of interest.
